# Do the methods used to analyse missing data really matter? An examination of data from an observational study of Intermediate Care patients

**DOI:** 10.1186/1756-0500-5-330

**Published:** 2012-06-27

**Authors:** Billingsley Kaambwa, Stirling Bryan, Lucinda Billingham

**Affiliations:** 1 Health Economics Unit, Public Health Building, University of Birmingham, Edgbaston, Birmingham, B15 2TT, United Kingdom; 2 Centre for Clinical Epidemiology and Evaluation, University of British Columbia, Research Pavilion 702-828 West 10th Ave, Vancouver, Canada; 3 Cancer Research UK Clinical Trials Unit (CRCTU), University of Birmingham, Edgbaston, Birmingham, B15 2TT, United Kingdom; 4 MRC Midland Hub for Trials Methodology Research, University of Birmingham, Edgbaston, Birmingham, B15 2TT, United Kingdom

**Keywords:** Missing data, Complete case analysis, Multiple imputation, Generalised linear model, Heckman selection, Observational data

## Abstract

**Background:**

Missing data is a common statistical problem in healthcare datasets from populations of older people. Some argue that arbitrarily assuming the mechanism responsible for the missingness and therefore the method for dealing with this missingness is not the best option—but is this always true? This paper explores what happens when extra information that suggests that a particular mechanism is responsible for missing data is disregarded and methods for dealing with the missing data are chosen arbitrarily.

Regression models based on 2,533 intermediate care (IC) patients from the largest evaluation of IC done and published in the UK to date were used to explain variation in costs, EQ-5D and Barthel index. Three methods for dealing with missingness were utilised, each assuming a different mechanism as being responsible for the missing data: complete case analysis (assuming missing completely at random—MCAR), multiple imputation (assuming missing at random—MAR) and Heckman selection model (assuming missing not at random—MNAR). Differences in results were gauged by examining the signs of coefficients as well as the sizes of both coefficients and associated standard errors.

**Results:**

Extra information strongly suggested that missing cost data were MCAR. The results show that MCAR and MAR-based methods yielded similar results with sizes of most coefficients and standard errors differing by less than 3.4% while those based on MNAR-methods were statistically different (up to 730% bigger). Significant variables in all regression models also had the same direction of influence on costs. All three mechanisms of missingness were shown to be potential causes of the missing EQ-5D and Barthel data. The method chosen to deal with missing data did not seem to have any significant effect on the results for these data as they led to broadly similar conclusions with sizes of coefficients and standard errors differing by less than 54% and 322%, respectively.

**Conclusions:**

Arbitrary selection of methods to deal with missing data should be avoided. Using extra information gathered during the data collection exercise about the cause of missingness to guide this selection would be more appropriate.

## Background

Missing data is an unwanted reality in most evaluations of services for older people as it can lead to biased results as well as threats to the generalisability and power of the results obtained from analysing such data [1,2]. Even under the best of conditions, missing data may result in a significant reduction in sample size leading to threats to external validity as a sample reduced in size may no longer be representative of the target population [3-5]. This is more problematic in circumstances where the likelihood of response is related to observed characteristics. Certain forms of missingness can reduce the statistical power of the analyses of the available data and therefore compromise the internal validity of a study, which is more serious [3,6,7]. A situation that can potentially lead to reduced statistical power is when the probability of response is associated with the values of the variable for which values are only partly observed, which is a possibility in a lot of cases [8].

The three main mechanisms that lead to missing data are: missing completely at random (MCAR), missing at random (MAR) and missing not at random (MNAR). If data are MAR or MCAR, they can also be referred to as “ignorable” data while those MNAR are “non-ignorable” [8]. Missing data are said to be ignorable if the parameters that are used to model the missing data process are not related to the parameters used to model the observed data while non-ignorability exists if there is a systematic difference between responders and nonresponders even after accounting for all the observed data [7,9]. There are various methods that have been proposed to deal with missing data with each of these methods premised on a specific missing data mechanism [1,10,11]. Croninger and Douglas [7] indicate that the choice of method used for coping with missing data is not crucial if there is not much missing data and/or the sample is big. This is because most methods will yield similar results in such circumstances. But as the level of missingness rises and/or the sample becomes smaller, the choice of method becomes potentially more significant. In this paper, we do not provide a detailed discussion of the various methods that can be used to deal with missing data. Interested readers can see Fielding et al. [12] for such a discussion. In general though, complete case analysis (both listwise and pairwise deletion) can be performed when data are MCAR [13]. Approaches for use when data are MAR include listwise deletion, various imputation techniques, propensity adjustment strategy, raw maximum likelihood and expectation maximisation [1,3,6,14]. When data are MNAR, panel selection models, including the Heckman, and pattern-mixture approaches can be used [15-17].

Most times, the method chosen to deal with missing data is not based on concrete evidence of the mechanism responsible for this missing data. It is consequently difficult to assess the accuracy of such methods because the data are by definition ‘missing’ [12]. It is a recognised fact that data often provide little or no information at all to help determine the correct mechanism behind missingness [3,18]. In many scenarios, therefore, it is difficult, or even impossible, to know what mechanism is responsible for the missingness. Sometimes more than one mechanism may be responsible for different sets of missing data within the same evaluation [7,19]. This therefore means that choosing among these alternative methods is not an easy task.

Curran et al. [19] suggest two approaches for determining the missing data mechanism: (1) hypothesis testing and (2) collecting extra information, during the data collection process, about why missing data is missing. In the absence of missing data being recovered and analysed, hypothesis testing can at best only rule out that missing data are MCAR with no way of confirming that data are actually MCAR [20]. Provided enough data has been collected, it therefore seems that, where missing data is irrecoverable, it is only the latter approach that will give some fairly credible indication about whether data are MCAR, MAR or MNAR [8,19].

This study explores what happens when extra information that suggests that a particular mechanism is responsible for missing data is disregarded and methods for dealing with the missing data are chosen arbitrarily. A dataset from the largest evaluation of intermediate care services done and published in the UK to date is used [21]. Intermediate services (IC) are tailored to prevent admission to acute care or long-term care and also aid discharge from hospital for older people [21]. It is not usual practice for such extra information to be gathered as part of the data collection process in a evaluation such as that for IC and the presence of this information therefore presented a unique opportunity to empirically compare different methods for dealing with missing data. As far as we are aware, this is the first time that this sort of analysis has been done on a dataset of older people in the UK. Using this dataset, which had missing data on several variables, the factors that explain variation in costs per patient, change in EQ-5D from admission to discharge (ΔEQ-5D) and change in the Barthel index from admission to discharge (ΔBarthel) of IC patients were explored in a regression modelling framework. These factors could be broadly divided into three groups: IC episode characteristics, descriptors of IC services and descriptors of IC-related services. Three methods incorporating techniques for dealing with missing data were used: (1) generalised linear models (GLMs) and ordinary least squares (OLS) on complete cases (assuming that missing data were MCAR), (2) GLM and OLS models on data obtained through multiple imputation (MI) (assuming missing data were MAR) and (3) Heckman selection models (assuming that missing data were MNAR). We were interested in examining the signs of coefficients as well as the sizes of both coefficients and associated standard errors in the regression model results obtained.

## Methods

### Source of data

Data for this study were obtained from five anonymous case study sites in the UK which were part of the National Evaluation of the Costs and Outcomes of IC for Older People (ICNET) [21]. These sites were ‘whole systems’ of IC i.e. areas with a specific geographical boundary. Quantitative data were collected by staff working for the IC services according to protocols set out by the evaluation team. Service staff completed study pro forma, with or on behalf of their patients, at the point of admission to the service, for all IC admissions over a defined period. They completed discharge questions on the day of discharge, transfer to another IC service or as soon as possible following end of service provision. In addition, extra information on the reasons as to why some data were missing was obtained from IC coordinators, ICNET researchers’ observations as well as from preliminary statistical analyses done on the ICNET dataset [21-23]. Data were collected between January 2003 and January 2004. Ethical approval was granted by the Trent Multicentre Research Ethics Committee.

### Missing data in the ICNET dataset

**Figure 1:**
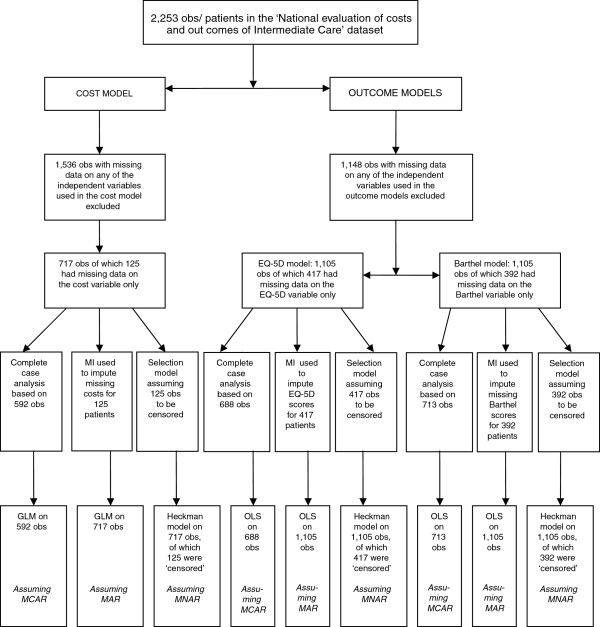
**Flow chart showing the data used in the analyses.** obs = observations; MI = multiple imputation; GLM = Generalised linear model; OLS - Ordinary least squares; MCAR = missing completely at random; MAR = missing at random; MNAR = missing not at random.

The variables that were collected in the ICNET dataset, based on a sample of 2,253 patients, are presented in Table 1. Up to 42% of the data were missing for some variables in that dataset. Extra information about why data were missing were available for all dependent variables (cost per patient, ΔEQ-5D and ΔBarthel) but not available for nearly all of the independent variables. Because of this lack of information and for purposes of comparing the methods for dealing with missing data, a decision was made to focus on missingness only in the dependent variables. Therefore, 1,536 out of 2,253 observations were excluded from the analyses reported in this paper due to missing values in the independent variables. There was therefore no missing value for all independent variables (and interaction terms generated using these variables) used in the analyses. A flow chart showing how the samples used in the final regression models were arrived at is shown in Figure 1. A sample of 717 individuals was therefore used for the cost per patient models and 125 (17.4%) of these individuals had missing observations on the cost variable. For the ΔEQ-5D and ΔBarthel models, a sample of 1105 individuals was utilised. Of this sample, 417 (37.7%) and 392 (35.5%) had missing values on the ΔEQ-5D and ΔBarthel variables, respectively.

**Table 1:** Variables for use in economic analysis (with level of completeness)

**Variable**	**Description**	**Missing (%)**
**Episode Characteristics**		
Age	Age on 01/01/03	3
Gender	1 = female , 0 = Male	2
Live alone	1 = Individual lives alone, 0 = Otherwise	9
Barthel – Start	Barthel Score at start of IC episode	31
Barthel – End	Barthel Score at end of IC episode	38
EQ5D – Start	EQ-5D at start of IC episode	40
EQ5D – End	EQ-5D at end of IC episode	41
Change in ED-5D	Difference between EQ-5D score at end and at start of IC episode	42
Change in Barthel	Difference between Barthel score at end and at start of IC episode	41
Cost	Cost per patient	38
**Descriptors of IC Services**		
	*Type of service required*	3
Admission Avoidance service	1 = Acute Admission Avoidance service, 0 = Otherwise	
Supported Discharge service	1 = Supported discharge service, 0 = Otherwise	
Other Service	1 = Other IC Services, 0 = Otherwise	
Type of IC	1 = Residential IC, 0 = Non-Residential IC	0
	*Outcome of IC episode*	13
Transfer	1 = Transferred before end of IC episode, 0 = Other outcome	
Complete	1 = Completed IC episode, 0 = Otherwise	
Died	1 = Patient Died, 0 = Otherwise	
Other Outcome	1 = Alternative Outcome, 0 = Other outcome	
Stay Duration	Duration of service provision (number of days)	17
**Descriptors of IC related services**		
	*Source of referral*	3
Referral – primary	0 = Otherwise, 1 = Primary Care	
Referral – hospital	0 = Otherwise, 1 = Hospital	
Referral – social	0 = Otherwise, 1 = Social Services	
Referral – other	0 = Otherwise, 1 = Other Sources	
	*Alternatives to IC services*	18
Alternative – Home	0 = Else, 1 = Home	
Alternative – Hospital	0 = Else, 1 = Hospital	
Alternative – other	0 = Else 1 = Other alternative	

### The dependent variables

The cost per patient variable was calculated by combining resource data with budget information for the individual IC services.

The EQ-5D is an outcome measure whose construct validity when used on populations of older people has been well documented [24-26]. It is comprised of five dimensions of health: mobility, self-care, usual activities, pain/discomfort, and anxiety/depression. There are three levels of impairment in each domain: no, some/moderate, and extreme problems in the relevant dimension of health. Using these responses, the EQ-5D is able to distinguish between 243 states of health [27,28]. The UK-specific EQ-5D valuation algorithm was used in order to convert the EQ-5D health description into a valuation. EQ-5D scores have a range of −0.59 to 1: the maximum score of 1 represents perfect health and a score of 0 represents death [28]. Scores less than 0 represent health states that are worse than death [28-30]. Its generic nature makes it comparable across patient populations.

The Barthel Index (BI) is a non-utility based conventional clinical scale of functional independence which has been recommended by the Royal College of Physicians for routine use in the assessment of older people [31]. Its validity when used on a general population of older people has also been shown [32] . To measure a person’s level of functional independence, the BI uses 10 items, with each item carrying different weights [33]. Two items (bathing and grooming) are rated on a two-point scale of 0 and 5, six (feeding, dressing, bowels, bladder, toilet use and stairs) on a three-point scale of 0, 5 and 10 and the last two items (transfers and mobility) are rated on a four-point scale of 0, 5, 10 and 15. The scores on each item are added to produce an overall score which ranges from 0 to 100. To standardise them, the overall scores used in this paper were divided by 5 and therefore ranged from 0 to 20 [34]. The higher the score recorded for an item, the greater the level of independence. The reliability, sensitivity and suitability for proxy-assessment of the BI has been shown elsewhere [33-35].

### Reasons for missing data in the ICNET dataset

When data are MCAR, it implies that the probability of an item missing is unrelated to any measured or unmeasured characteristic for that unit [36], while under MAR, the probability of an item having incomplete data depends on other variables in the dataset [1]. MNAR is when the probability of missingness depends on the values of the unobserved values perhaps in addition to one or more other variables and/or the observed variables [37].

Because of time constraints placed on the data collection process, it was not possible to collect all of the cost data. No other reason was established as being responsible for the missing cost data. This suggests that where cost data were missing, it would be reasonable to assume that these data were MCAR.

In terms of the missing data on the EQ-5D and Barthel, all three mechanisms (MCAR, MAR and MNAR) could be assumed as the reason for this missingness.

Firstly, information obtained from the IC coordinators about some of the missing EQ-5D and Barthel data indicated that some services did not routinely collect this information while some of the item non-responses were ascribed to administrative errors [21]. This suggested that it was plausible to assume that the missingness mechanism for such data was MCAR.

Secondly, the ΔEQ-5D and ΔBarthel scores were calculated by subtracting the scores at admission from those at discharge. A number of individuals had however been transferred to other services before the end of their IC episode. For some of these, it meant that their EQ-5D and Barthel scores at ‘discharge’ were not collected making it impossible to compute the ΔEQ-5D and ΔBarthel variables. This could be seen as a situation where the missing data were MAR as the reason for the patients transfer was more often than not linked to their health or functional status e.g. the more functionally independent an individual was, the more likely they were to be transferred to a less intensive form of IC. Additional statistical analyses on the IC dataset [23] also revealed that the Barthel scores were predictive of the missing EQ-5D values, further reinforcing the plausibility of the missing EQ-5D data being MAR.

Thirdly, the mean Barthel scores for some individuals who had missing EQ-5D scores were on average lower than those for individuals who did not have missing EQ-5D information [22]. Since some individuals with missing EQ-5D data were associated with lower Barthel scores, it means that, by virtue of the positive relationship between the two instruments [23], there is a possibility that these individuals would also have had lower EQ-5D scores had these been collected. It was therefore reasonable to assume that some of the missing data on the EQ-5D could also have been MNAR i.e. the poorer ones’ health status was, the more difficult it was for them to provide data on the EQ-5D. By the same token, some of the missing Barthel data could have been MNAR.

### Choice of regression families

In this exercise, it was important to compare both the signs and sizes of coefficients (and sizes of associated standard errors) from the different regression models. Both costs per patient and outcome variables were skewed and heteroscedastic in their residuals. We chose the GLM as it is able to simultaneously deal with both problems [38,39]. We also used log-transformation where the natural log of the dependent variable was obtained [40] as another method for dealing with the skewed cost data despite several limitations associated with this approach [41,42]. For the cost models, therefore, a decision was made for the GLM to be used for both the complete cases and the multiply imputed datasets while a log transformed cost per patient was used in the Heckman regression model. As the exponentiated coefficients from the GLM model have been shown to be easily comparable to the exponentiated counterparts obtained from a log-transformed model [43], the results of all the cost per patient models are presented in terms of exponentiated coefficients.

A different approach was taken for the health-outcome dependent variables (ΔEQ-5D and ΔBarthel). This was because these variables also had negative values. As a result, log transformation of these variables would have required the use of a shift factor and the transformed variables would then have had to be appropriately retransformed once the results of the model had been obtained. However for ease of analysis and comparison, a decision was made to use the raw scale of these variables. As a result, OLS regressions were used for both the ΔEQ-5D and ΔBarthel in the regression on complete cases and on multiply imputed datasets. Further, OLS regressions on a raw scale have also been widely used for modelling such outcome data in the literature [44]. The raw scale of the two variables was also used in the Heckman selection models.

### Approaches for dealing with the missing data

For our two samples (n = 717 and n = 1,105) obtained from the ICNET dataset, three methods, each assuming either MCAR, MAR or MNAR, were used. A regression framework was employed in the analysis and in general, the regression relationship between the outcomes of interest and the independent variables could be illustrated as [45]:

(1) $${\text{Y}}_{i}=\beta_{0}+\beta_{1i}+\text{…}+\beta_{k}+X_{ki}+\mu_{i}$$

where *Y*_*i*_ denotes the outcome of interest (cost per patient, ΔEQ-5D or ΔBarthel) for the ith individual, *β*_*i*_ … _*k*_ are the coefficients, *X*_*i*_ … _*k*_ are the explanatory variables (both single and interaction terms) for the ith individual and *μ*_*i*_ is the stochastic error term for the ith individual.

A total of six sets of regression models (two for each method) were conducted:

Method 1 involved running regression models on complete cases (assuming that data were MCAR). A GLM was used to explain variation in ‘cost per patient’ while OLS models were run for cases where the dependent variables were ΔEQ-5D and ΔBarthel. Pairwise deletion, implying the use of all available data on the particular variables specified in each model, was the method used to arrive at the samples modelled as complete cases, As a result, disparate sample sizes of 592, 688 and 713 observations for the cost per patient, ΔEQ-5D and ΔBarthel models, respectively, were used (please see Figure 1).

Method 2 involved running GLM and OLS regression models again to explain variation in costs per patient and outcomes (ΔEQ-5D and ΔBarthel), respectively. Here, however, we used multiply imputed datasets (assuming that data were MAR) based on a multivariate normal model. [1] Up to about 38% of the data were missing and multiply imputed datasets were created to account for these missing data before running GLM and OLS regression models. These analyses focussed on imputing values for the dependent variables where the independent variables were not missing thereby creating complete datasets i.e. 717 observations for the cost per patient model and 1105 observations for the ΔEQ-5D and ΔBarthel models. The rationale for this particular imputation was to allow for direct comparison between the results obtained using this method and those produced by method 3 (described below), which comparison required that essentially the same samples were analysed. Five sets of imputations were created following conventional practice [11]. Since there was up to 38% data missing, these imputations led to point estimates that were at least (1 + 0.38/5)^−1^ = 93% as efficient as those based on m = ∞ imputations [1].

In method 3, Heckman selection models (assuming that missing data were MNAR) were run on the log of ‘cost per patient’, on ΔEQ-5D and ΔBarthel using ‘complete cases’. Whereas method 1 only considered cases where there was no missing data for both the dependent variable and independent variables, method 3 considers all subjects including those that had missing cost, EQ-5D or Barthel information. The sample selection used a dummy variable equal to 1 if the dependent variable was not missing and equal to 0 if it was. Using this classification, 125 out of 717 observations were censored (missing) for the cost per patient model while 417 and 392, out of 1105 observations, were censored for the ΔEQ-5D and ΔBarthel models, respectively.

Multiple imputations were conducted in NORM [46] while the rest of the analyses were done in STATA version 8.2 [47].

## Results

The results of the above analyses are presented in Table 2 for the costs per patient models and Tables 3 and 4 for the ΔEQ-5D and ΔBarthel models, respectively.

**Table 2:** Comparison of results from three methods of regression analysis of costs per patient

		***GLM on complete cases n = 592*[**[1]**]**^***a***^		***GLM on MI dataset n = 717*[**[2]**]**^***b***^		***Heckman on complete cases n = 717, 125 obs censored*[**[3]**]**^***c***^	
	**Variables**	**Exp (Coeff)**	**S.E.**	**Exp (Coeff)**	**S.E.**	**Exp (Coeff)**	**S.E.**
Episode Characteristics	Age in 2003	0.996	0.003	0.997	0.002	1.000	0.012
	Gender	0.982	0.063	1.009	0.060	1.085	0.281
	Lives alone	1.052	0.059	1.047	0.056	1.106	0.275
	Barthel score at admission	0.973	0.009**	0.984	0.008*	0.884	0.061*
	EQ5D score at admission	0.973	0.090	0.935	0.087	1.400	0.435
Descriptors of IC Service	Acute Admission Avoidance Service	0.930	0.129	0.812	0.092*	6.723	0.960*
	Type of IC	3.181	0.079**	3.150	0.070**	5.146	1.274
	Transferred before end of IC episode	1.144	0.310	1.259	0.258	1.316	1.422
	Completed IC episode	2.094	0.300*	2.396	0.248**	4.611	1.318
	Other IC Outcome	2.703	0.337**	2.796	0.287**	4.374	1.475
	Patient Died (Reference. Group)						
Descriptorsof IC-related Services	Referral – Primary	0.777	0.123*	0.764	0.121*	0.936	0.576
	Referral – Hospital	0.914	0.158	0.777	0.134	4.523	0.930
	Referral – Other	1.001	0.212	0.935	0.195	2.240	0.984
	Referral – Social Workers (Reference Group)						
	Alternative to IC – Other	1.053	0.079	1.058	0.077	0.508	0.451
	Alternative to IC – Home	1.121	0.074	1.058	0.070	1.112	0.329
	Alternative to IC – Hospital (Reference Group)						
Interactions	Barthel score at admission*Type of IC	1.031	0.018	1.017	0.097	1.131	0.092
	Acute Admission Avoidance Service* Type of IC	1.214	0.163	1.217	0.136	0.579	0.752
	Transfer before IC end*Type of IC	1.145	0.185	1.176	0.169	1.145	0.825
	Completed Episode*Type of IC	1.152	0.195	1.112	0.162	0.240	0.952
	Other IC Outcome*Type of IC	0.717	0.708	0.583	0.534	0.773	2.846
	Patient died*Type of IC (Reference group)						
	_constant	1140.3	0.421**	951.5	0.360**	345.3	1.866**
	N		592	717			717
	Censored obs						125
	R-Squared		0.359				0.634
	Rho						0.950

* 5% level of significance, ** 1% level of significance; Dependent variable: cost per patient for GLM and log of cost per patient for Heckman Selection model;

IC = Intermediate care; ^a^Method 1 assumes that missing data are MCAR;

^b^Method 2 assumes that missing data are MAR; ^c^Method 3 assumes that missing data are MNAR.

**Table 3:** Comparison of results from three methods of regression analysis (Change in EQ5D)

		**OLS on complete cases n = 688 [1]^a^**		**OLS on MI dataset n = 1105 cases [2]^b^**		**Heckman on complete cases n = 1105, 417 obs censored [3]^c^**	
	**Variables**	**Coeff**	**S.E.**	**Coeff**	**S.E.**	**Coeff**	**S.E.**
Episode Characteristics	Age in 2003	0.000	0.001	0.000	0.001	0.000	0.001
	Gender	0.046	0.022*	0.051	0.018**	0.054	0.024*
	Lives alone	0.020	0.020	0.015	0.017	0.029	0.023
	Barthel score at admission	0.017	0.003**	0.017	0.002**	0.016	0.003**
	EQ5D score at admission	−0.495	0.033**	−0.479	0.026**	−0.484	0.037**
Descriptors of IC Service	Acute Admission Avoidance Service	−0.038	0.027	−0.017	0.021	0.156	0.042**
	Duration of Service Provision	0.000	0.000	0.001	0.000*	0.000	0.000
Descriptors of IC-related Services	Referral – Primary	−0.031	0.052	−0.044	0.043	−0.020	0.058
	Referral – Hospital	−0.098	0.051	−0.053	0.042	0.020	0.059
	Referral – Other	−0.003	0.078	0.059	0.065	0.013	0.086
	Referral – Social Workers (Reference Group)						
	Alternative to IC – Other	−0.063	0.031*	−0.077	0.025**	−0.077	0.030*
	Alternative to IC – Home	−0.045	0.023*	−0.028	0.019	−0.046	0.022*
	Alternative to IC – Hospital (Reference Group)						
Interactions	Gender*Type of IC	−0.048	0.053	−0.027	0.037	−0.057	0.053
	Barthel score at admission*Type of IC	0.003	0.004	−0.002	0.003	0.004	0.004
	EQ5D score at admission *Type of IC	−0.098	0.083	0.061	0.057	−0.118	0.082
	Acute Admission Avoidance Service*Type of IC	0.110	0.064	0.039	0.039	0.086	0.063
	Alternative to IC – Other *Type of IC	0.137	0.084	0.133	0.059*	0.140	0.082
	Alternative to IC – Home*Type of IC	0.086	0.106	−0.027	0.049	0.070	0.104
	Alternative to IC – Hospital *Type of IC (Reference Group)						
	_constant	0.157	0.101	0.093	0.084	0.100	0.105
	N		688	1,105			688
	Censored obs						417
	R-Squared		0.284	0.266			0.634
	Rho						0.950

* 5% level of significance, ** 1% level of significance;

Dependent variable: change in EQ-5D, IC = Intermediate care

^a^Method 1 assumes that missing data are MCAR; ^b^Method 2 assumes that missing data are MAR; ^c^Method 3 assumes that missing data are MNAR.

**Table 4:** Comparison of results from three methods of regression analysis (Change in Barthel)

		**OLS on complete cases n = 688 [1]^a^**		**OLS on MI dataset n = 1105 cases [2]^b^**		**Heckman on complete cases n = 1105, 417 obs censored [3]^c^**	
	**Variables**	**Coeff**	**S.E.**	**Coeff**	**S.E.**	**Coeff**	**S.E.**
Episode Characteristics	Age in 2003	−0.010	0.009	−0.009	0.007	−0.011	0.009
	Gender	−0.007	0.208	0.097	0.164	0.037	0.218
	Lives alone	0.225	0.190	0.181	0.150	0.320	0.202
	Barthel score at admission	−0.318	0.028**	−0.325	0.022**	−0.305	0.030**
	EQ5D score at admission	−0.343	0.312	−0.428	0.239	−0.216	0.328
Descriptors of IC Service	Acute Admission Avoidance Service	0.103	0.218	0.060	0.167	0.728	0.337*
	Duration of Service Provision	0.008	0.003*	0.011	0.003**	0.006	0.003*
Descriptors of IC-related Services	Transfer before IC end	4.084	2.452	0.559	0.607	2.713	2.348
	Completed Episode	7.438	2.440**	3.443	0.587**	4.926	2.478*
	Other IC Outcome	6.640	2.477**	2.921	0.656**	4.727	2.432
	Patient died (Reference group)						
	Alternative to IC – Other	−1.130	0.291**	−1.076	0.221**	−1.267	0.291**
	Alternative to IC – Home	−0.709	0.223**	−0.667	0.169**	−0.669	0.219**
	Alternative to IC – Hospital (Reference Group)						
Interactions	Barthel score at admission*Type of IC	−0.071	0.050	−0.035	0.027	−0.072	0.051
	Acute Admission Avoidance Service* Type of IC	0.592	0.575	0.131	0.354	0.599	0.589
	Duration of Service Provision*Type of IC	−0.006	0.008	0.001	0.006	−0.006	0.008
	Transfer before IC end*Type of IC	−0.299	0.979	−0.160	0.411	−0.300	0.962
	Completed Episode*Type of IC	1.053	0.830	0.374	0.424	1.055	0.816
	Other IC Outcome*Type of IC	0.189	2.000	0.072	0.889	0.200	1.980
	Patient died*Type of IC (Reference group)						
	Alternative to IC – Other *Type of IC	0.796	0.793	0.968	0.543	0.795	0.778
	Alternative to IC – Home*Type of IC	2.261	1.025*	0.124	0.447	2.261	1.006*
	Alternative to IC – Hospital *Type of IC (Reference Group)						
	_constant	0.046	2.536	3.888	0.843	2.687	2.592
	N		713	1,105			713
	Censored obs						392
	R-Squared		0.278				0.634
	Rho						0.950

*5% level of significance, **1% level of significance;

Dependent variable: change in Barthel, IC = Intermediate care;

^a^Method 1 assumes that missing data are MCAR; ^b^Method 2 assumes that missing data are MAR; ^c^Method 3 assumes that missing data are MNAR.

### Cost per patient models

The results of the GLM regression model on complete cases (method 1) and GLM regression model on multiply imputed datasets (method 2) are similar. As shown in Table 2, significant predictors of cost per patient were the Barthel score at admission, IC function (acute admission avoidance service or not), type of IC (residential or not), if one completed an IC episode, other IC outcome and if the source of referral was primary care. All of the variables that were found to be significant in method (2) were also significant in method (1) with the exception of one (acute admission avoidance service) which was significant in model (2) only. Also, the size of coefficients for nearly all of these variables differed by less than 3.4% except the one for ‘completed IC episode’ which differed by about 14.4%. The sizes of the standard errors were also similar. Further, the variables significant in both models had the same direction of influence on costs per patient. On the other hand, the results obtained from the Heckman selection regression model (method 3) were much more different. A lot more variables were found to be insignificant with only two variables (Barthel score at admission and acute admission avoidance service) shown to significantly influence costs per patient. The sizes of the coefficients in the Heckman model were also different from those of the other two methods. For instance, the coefficient for ‘acute admission avoidance service’ was about 730 times bigger than that obtained in method (2). The mills ratios were −3.402 and −4.506 for the Heckman selection models with and without interactions, respectively. These were both statistically significant at 95% level of significance.

### Change in EQ-5D models

Here, the results from all three models/methods were broadly similar (Table 3). Significant predictors of ΔEQ-5D were gender, Barthel score at admission, EQ-5D score at admission, IC function, duration of service provision and likely alternatives were IC not available (home and other alternative). Nearly all of the variables that were significant in one model were also significant in the other models. The only exceptions were the ‘duration of service provision’ and ‘Alternative to IC-Other*Type of IC’ (both only significant in method 2), ‘acute admission avoidance service’ (only significant in method 3) and ‘alternative to IC-Other’ (significant only in models 1 and 3). The sizes of the coefficients of variables commonly significant in all models differed at most by about 22% with the standard errors differing at most by 42% (Table 3). Further, the variables significant in all three models had the same direction of influence on the change in EQ-5D. The mills ratios were −0.284 and −0.143 for the Heckman selection models with and without interactions, respectively. These were both statistically significant at 95% level of significance.

### Change in Barthel models

As in the ‘change in EQ-5D’ models, the results obtained from all three models/methods for the change in Barthel were broadly similar (Table 4). Significant predictors of ΔBarthel were the Barthel score at admission, IC function, outcome of IC episode (completed and other), likely alternatives were IC not available (home and other) and an interaction term between likely alternatives were IC not available and type of IC. All of the variables that were significant in one model were also significant in the other models with the exception of ‘acute admission avoidance service’ and ‘Alternative to IC—Home*Type of IC’ (only significant in method 3) and ‘Other IC Outcome’ variable only significant in both method (1) and method (2). However, the differences in terms of the sizes of coefficients and standard errors of variables significant in all methods were slightly bigger in these models than in the ‘change in EQ-5D’ models. They differed at most by about 54% and 322% for coefficients and standard errors, respectively. The variables significant in all three models had the same direction of influence on the change in Barthel. The mills ratios were −1.662 and −0.101 for the Heckman selection models with and without interactions, respectively. These were both statistically significant at 95% level of significance.

## Discussion

The ICNET dataset had up to 42% and 38% of the data on EQ-5D and Barthel scores, respectively, missing while 31% of the sample had missing cost data. In terms of other variables in the dataset, all but one (type of IC) had missing data ranging from 3 to 18%. This situation is common to a vast number of health service research datasets for older people. If these missing data are simply ignored, then there is a chance that biased and underpowered results may be obtained [1,48]. The most appropriate method of dealing with this amount of missingness therefore had to be determined [19,49]. The results of this analysis have shown that, in determining the methods to deal with missing data, using extra information gathered during the data collection exercise about the cause of missingness, rather than the arbitrary selection of such methods, is more appropriate. There is however need to carry out similar analyses in datasets based on individuals with different characteristics in order to discount the effect that attributes specific to this dataset, such as the age of respondents, may have had on these results.

The evidence gathered concerning the missing cost data strongly suggested MCAR as the reason for this missingness. When MAR-based methods were used for these data, the results obtained were not significantly different from those based on the MCAR assumption. These findings seem to bear out the position held by Schafer and Graham [50] and David et al. [51] that in many realistic applications, departures from MAR are not big enough to effectively invalidate the results of an MAR-based analysis. A similar position was arrived at by Foster and Fang [8]who found that estimates based on listwise deletion (assuming MCAR) and those based on MI and ignorable maximum likelihood estimation (both assuming MAR) were comparable. The use of an MNAR-based method in the costs per patient model yielded results that were so different to those obtained when either MCAR or MAR were assumed. In particular, fewer significant variables were obtained in the MNAR-based method while, similar to the study by Foster and Fang [8], the sizes of the coefficients were larger. Therefore, different conclusions could potentially be reached if the MNAR assumption was made for the missing cost data. Care must therefore be taken not to apply MNAR-based methods when it is not absolutely clear that the missing data are MNAR as MNAR approaches often require assumptions that cannot be validated from the data at hand [52]. MNAR-based approaches are best implemented as sensitivity analyses so as to assess how robust results are across different analytic approaches [53].

All three mechanisms of missingness were shown to be potential causes of the missing EQ-5D and Barthel data. When observations in the dependent variable are MAR while the independent variables are complete, Little [54] posits that the incomplete cases contribute no information to the regression where such a dependent variable is modelled. While some, as a consequence, have deleted cases with missing values on the dependent variable, which approach effectively reduces to a complete case (regression) analysis [55], others have used imputed values of the dependent variable in subsequent regression analyses [16]. In this study, we did both despite the fact that we did not have outcome data that were purely MAR. The results from the ΔEQ-5D and ΔBarthel models show that the choice of mechanism did not have a very significant effect on the results. Despite the sizes of the coefficients and standard errors being somewhat different, the results from all three methods were broadly comparable in that similar conclusions could have been reached on the back of running the models. A possible explanation for this may have been the fact that the reason for missing data could be ascribed to any one of the three mechanisms of missingness or indeed a combination of these mechanisms. Croninger and Douglas [7] also assert that MCAR and MAR-based methods are relatively robust if the sample size is modestly large even when missing data are MNAR. While the extra information gathered during the data collection process supported the assertion that the missing data were either MCAR, MAR or MNAR, the significant mills ratios lent additional support to the MNAR assumption as its significance in the selection models indicated the presence of significant selection bias. However, selection models, even though identifiable, should be treated with caution especially when data are possibly not MNAR [56].

In this study, there were limitations in terms of accurately determining the reasons for the missing data as this determination relied on the views of IC coordinators, investigators’ observations and some statistical analyses carried out on the ICNET dataset. Determinations based on this extra information were not definitive. Further, this information was only available for dependent variables. A more formal way of collecting this extra information may include adding questions, within the main data collection instrument, about why these data are missing and this should be done for both dependent and independent variables. A critical evaluation of the responses to these questions will help inform the process of identifying the missingness mechanism. In the absence of hypothesis testing, however, this extra information provided the best insights into why the missing data were not collected. In addition, the exclusion of missing observations in the independent variable may have altered the missing data mechanisms. This however would mainly apply to cases where data were MAR. As the MAR mechanism was premised mainly on EQ-5D and Barthel for which the missing observations were kept as low as possible, the probability of alterations in the missingness mechanisms was minimised. Finally, the use of untransformed OLS models for ΔEQ-5D and ΔBarthel in the presence of the skewed nature of the two variables could have potentially led to biased results. Tests of skewness performed on the variables have however showed low level of skewness (p values from the Shapiro-Wilk test for ΔEQ-5D and ΔBarthel were 0.047 and 0.042, respectively) implying that any bias resulting from the use of untransformed OLS models would also be minimal.

## Conclusions

Many studies have emphasised the importance of determining the mechanism behind missing data before deciding on the technique to use [19,49,57]. This paper considered three different mechanisms that may be responsible for missing data and then discussed approaches that can be used to deal with the missing data. The results from this analysis suggest that the methods used to analyse missing data really do matter especially when one is considering whether or not to use MNAR-based methods. Dealing with missing data is not easy especially as the hypothesis-based techniques for detecting the pattern of missingness are limited in that they can only be used to rule out MCAR but can not confirm this mechanism. Further, there are no hypothesis-test-based techniques available for determining if data are MAR or MNAR in cases where the missing data are irrecoverable. This therefore means that there should not be any arbitrary selection of assumptions behind data missing mechanisms and using extra information gathered during the data collection exercise about the cause of missingness to guide this selection would be more appropriate. In the absence of this extra information, then one of the MAR-based methods could be considered as these were shown in this study and elsewhere to be robust for use even in cases where data are strictly not MAR.

## Competing interests

The authors declare that they have no competing interests.

## Authors’ contributions

BK undertook the econometric analyses and wrote the first draft of the paper. Subsequent drafts were contributed to by SB and LB who have approved the final version. BK will act as guarantor. All authors read and approved the final manuscript.
